# RISE-VIO: Robust Initialization and Targeted Pose Robustification for INS-Centric Visual–Inertial Odometry Under Degraded Visual Conditions

**DOI:** 10.3390/s26082305

**Published:** 2026-04-08

**Authors:** Xiaowei Xu, Ran Ju, Wenhua Jiao, Lijuan Li

**Affiliations:** College of Electrical Engineering and Control Science, Nanjing Tech University, Nanjing 211800, China

**Keywords:** graduated non-convexity, outlier rejection, robust estimation, initialization observability, visual–inertial odometry

## Abstract

Feature-based visual–inertial odometry (VIO) often suffers from initialization failures and tracking drift under degraded visual conditions, such as low-texture regions, abrupt illumination changes, and scenes with a high ratio of dynamic correspondences. We present RISE-VIO, a real-time inertial-navigation-system-centric (INS-centric) visual–inertial odometry system that improves robustness by introducing GNC-style robustification into two failure-critical stages: initialization and per-frame pose estimation. For robust initialization, we develop a GNC-based decoupled rotation–translation initialization module with a two-stage observability gate, consisting of (i) rotation-compensated parallax-rate screening and (ii) a spectral-stability test on the linear global translation (LiGT) system. For online robustness, we design an IMU-prior-guided GNC-EPnP module to selectively downweight or reject outlier correspondences during pose estimation. Experiments on public benchmark datasets show that RISE-VIO achieves more reliable initialization and more stable trajectory estimation in challenging visual conditions while maintaining real-time performance. Additional Monte Carlo perspective-n-point (PnP) evaluations further support the robustness of the proposed pose estimation module under severe outlier contamination.

## 1. Introduction

Despite their maturity, feature-based visual–inertial odometry (VIO) systems frequently fail to initialize or maintain accurate pose estimates in practical conditions such as low-texture corridors, abrupt illumination transitions (e.g., entering/exiting tunnels), or scenes dominated by large moving objects. In these situations, sparse correspondences become unreliable and spurious matches proliferate, producing ill-conditioned estimation problems that trigger premature initialization or unstable tracking in representative pipelines (e.g., VINS-Mono and ORB-SLAM3) [1,2].

Two practical bottlenecks repeatedly constrain visual–inertial odometry in real deployments. First, initialization under weak motion excitation can cause incorrect separation between gravity and accelerometer bias, producing biased state estimates and poor downstream performance [3]. Second, optical-flow and correspondence outliers—caused by moving objects, occlusion, or transient lighting—corrupt pose estimation and landmark triangulation. Importantly, these issues are coupled: uncertain early bias/gravity can amplify the impact of mismatches, while early mismatches can push the initializer into numerically fragile configurations that worsen observability. Recent studies have explored complementary directions to improve robustness, including more efficient or decoupled visual–inertial initialization [4,5,6,7], and robustness-oriented VIO systems for dynamic or visually challenging environments [8,9,10,11]. These advances highlight both the importance of reliable initialization and the continuing need for lightweight outlier-tolerant pose estimation in real-time VIO.

To address these issues, we develop RISE-VIO, an inertial-navigation-system-centric (INS-centric) real-time visual–inertial odometry system for degraded visual conditions. Rather than proposing a new robust estimation theory or redesigning the entire backend, RISE-VIO improves robustness through targeted modifications to two failure-critical estimation stages in INS-centric VIO: startup initialization and per-frame pose estimation. Specifically, we develop a GNC-enhanced rotation–translation decoupled initializer with a two-stage observability test to prevent premature alignment under weak excitation, and an IMU-prior-guided GNC-EPnP module to suppress optical-flow mismatches during per-frame pose estimation.

Our contributions are summarized as follows:

- A lightweight and system-compatible integration of graduated non-convexity (GNC) into two failure-critical stages of INS-centric VIO—startup initialization and per-frame pose estimation—without reformulating the full backend as a robust optimization problem.
- A GNC-robustified decoupled initialization module for feature-based VIO, combining gyroscope-bias estimation with a two-stage observability test to improve startup reliability under weak excitation and degraded tracking.
- An IMU-prior-guided GNC-EPnP module for robust per-frame pose estimation, improving tolerance to correspondence outliers and dynamic interference while preserving the efficiency of EPnP.

The remainder of this paper is organized as follows. Section 2 reviews related work. Section 3 presents the overall system overview of RISE-VIO. Section 4 details the proposed method, including the notation and modeling assumptions, a brief mathematical primer on the GNC framework, the GNC-based robust initialization strategy, and the hierarchical outlier-rejection pipeline for robust tracking. Section 5 reports the experimental results and ablation studies. Finally, Section 6 concludes the paper.

## 2. Related Work

Early works on visual–inertial initialization pursued closed-form or analytical solutions, but typically relied on restrictive assumptions such as partially known inertial biases, limiting robustness across runs and sensor conditions. Subsequent approaches shifted toward nonlinear optimization to jointly estimate biases together with gravity and velocity, improving generality but still relying on iterative procedures that are sensitive to seeding and early tracking quality [3,12,13]. Later refinements emphasized practical robustness and speed within modern SLAM/VIO pipelines, yet the dependency on iterative alignment and sufficiently reliable feature tracks remains. More recently, alternative formulations have been proposed to improve efficiency and robustness by exploiting structural decompositions or stronger geometric constraints. In particular, rotation–translation decoupled initialization estimates rotation-related quantities (e.g., gyroscope bias) first and then solves translation/scale more efficiently, enabling faster bootstrapping while still becoming fragile under weak excitation or correspondence contamination [4]. Along this line, EDI introduced an ESKF-based disjoint initialization strategy to improve efficiency and practical robustness in visual–inertial SLAM pipelines [5]. Normal-epipolar-constraint-based variants further strengthened initialization by improving geometric consistency during early-stage estimation, including stereo initialization with NEC constraints [6] and recent methods that directly refine extrinsic orientation and gyroscope bias during VIO initialization [7]. Hybrid strategies can also recover both biases through filtering-style designs, at the expense of additional algorithmic complexity [14]. In parallel, uncertainty-aware modeling has strengthened initialization: PNEC explicitly accounts for feature uncertainty and has been adopted to improve gyroscope-bias estimation when measurements are noisy or partially corrupted [15].

Dynamic environments further amplify these challenges by injecting structured and persistent outliers into optical flow and data association. Semantic masking pipelines explicitly detect and suppress moving objects [16,17,18], but their reliance on heavyweight perception components may hinder real-time deployment on resource-constrained platforms [19]. Complementary non-learning, geometry-driven approaches reject dynamic outliers by enforcing motion consistency or modeling the static background, including static-point weighting and correlation/consistency screening [20,21], rigid-motion/background-model separation [22], and dense background reconstruction pipelines such as StaticFusion and ReFusion [14,23]. While effective, these geometric defenses often depend on explicit selection rules, auxiliary assumptions (e.g., depth/rigid foreground), or scene-dependent thresholds, and may degrade when outliers are intermittent, highly structured, or visually subtle. More recent VIO systems have also explicitly targeted robustness in dynamic or visually challenging conditions. For example, RD-VIO improves robustness in dynamic environments through IMU-guided tracking and deferred triangulation [8], while DynaVINS++ combines adaptive truncated least squares with stable state recovery to mitigate failures caused by dominant moving objects [9]. Recent works have further addressed challenging sensing conditions such as dynamic illumination and weak visual structure, including tightly coupled VIO with robust feature association under dynamic illumination [10] and line-feature-enhanced VIO for illumination-changing environments [11]. Recent engineering-oriented systems have also emphasized practical robustness and accuracy in challenging environments, including enhanced VIO pipelines for challenging flight conditions [24] and semantic-geometric systems for dynamic environments that combine lightweight object detection with adaptive geometric constraints [25].

Robust estimation provides a complementary route by embedding outlier suppression into optimization. Classical RANSAC remains widely used [26] but can struggle under high outlier ratios or correlated outliers induced by dominant movers. Graduated non-convexity (GNC) offers a principled mechanism to progressively downweight outliers and enables robust estimation with reduced sensitivity to initialization [27]. However, applying robustification broadly to full back-ends or dynamic visual–inertial SLAM systems can incur substantial computational overhead (e.g., DynaVINS and its subsequent robustness-oriented extensions) [9,28]. These observations motivate a lightweight, targeted use of robustification in the two stages that most directly affect system stability—startup initialization and per-frame pose estimation—while keeping the rest of the pipeline unchanged.

Existing selective or robust optimization strategies in SLAM/VIO are often introduced at the backend level, such as keyframe refinement, loop-closure optimization, or selective re-optimization of global constraints. In contrast, our work targets two local but failure-critical estimation modules in feature-based VIO: gyroscope-bias estimation during initialization and EPnP-based visual pose estimation. The contribution of this work therefore lies not in proposing a new robust estimation framework itself, but in identifying these two bottlenecks and integrating GNC-based robustification into them in a lightweight and system-compatible manner.

## 3. System Overview

**Figure 1:**
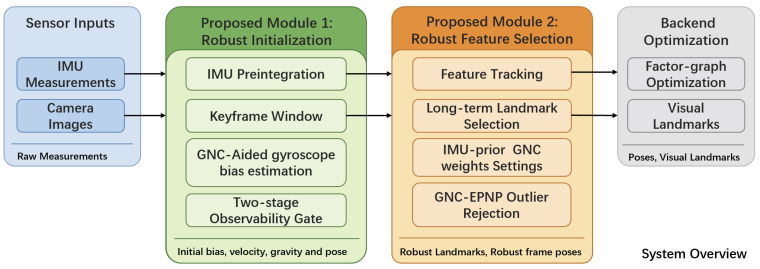
System overview of RISE-VIO. The proposed system augments a standard INS-centric visual–inertial pipeline with two modules introduced in this work, highlighted in the figure: robust initialization and robust feature selection before backend optimization. The remaining frontend tracking, IMU propagation, and backend factor-graph optimization follow a standard pipeline structure.

To address the above limitations, we develop RISE-VIO, an INS-centric visual–inertial odometry system designed for degraded visual conditions. As shown in Figure 1, the proposed system preserves the basic backend optimization structure of a standard visual–inertial pipeline, while introducing robustness only in two stages that are especially vulnerable to degraded visual measurements: startup initialization and frontend feature selection before pose estimation. This targeted design improves robustness without requiring a full redesign of the backend.

**Figure 2:**
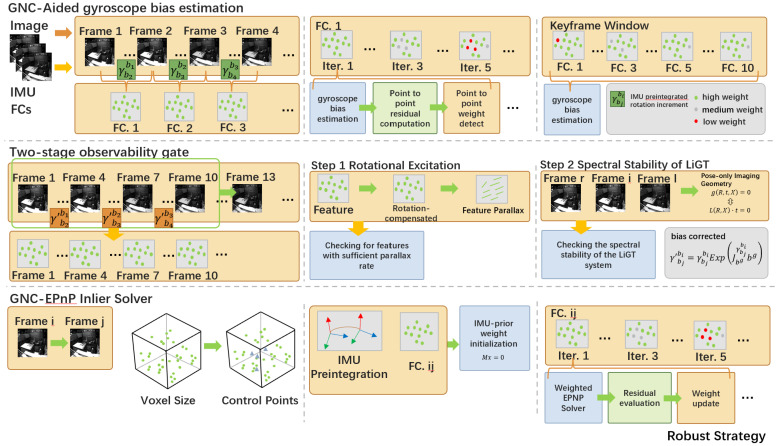
Detailed robust strategy in RISE-VIO. (**Top**): GNC-aided gyroscope bias estimation and keyframe-window construction for robust initialization. (**Middle**): two-stage observability gate for checking rotational excitation and spectral stability before initialization. (**Bottom**): IMU-prior-guided GNC-EPnP inlier solver for robust per-frame feature selection and pose estimation.

The first proposed module is a robust initialization strategy. During startup, IMU measurements are continuously integrated to provide short-term motion priors, while camera images are organized into a keyframe window for visual–inertial alignment. As illustrated in Figure 2, initialization is activated only after a two-stage observability gate verifies sufficient rotational excitation and acceptable spectral stability. Under this condition, a GNC-aided gyroscope bias estimation procedure is used to improve the reliability of the initial bias, gravity, velocity, and pose estimates.

The second proposed module is a robust feature-selection strategy for per-frame pose estimation. After initialization, the frontend tracks image features and selects long-term landmarks that are suitable for geometric pose recovery. Before the estimated frame pose is sent to the backend, an IMU-prior-guided GNC-EPnP module evaluates the consistency of 2D–3D correspondences, progressively downweights unreliable matches, and rejects outliers caused by degraded tracking or dynamic interference. The resulting robust pose estimate and landmark set are then fused in the backend factor-graph optimization.

In this way, RISE-VIO strengthens the two stages where degraded visual input most directly threatens system stability: initialization and per-frame visual update. The robust initialization strategy and the IMU-prior-guided pose estimation module are detailed in Section 4.3 and Section 4.4, respectively.

## 4. Method

### 4.1. Notation and Assumptions

All vectors and matrices are expressed in a right-handed Cartesian coordinate system. Vectors are denoted by bold lower-case letters (e.g., x), unit vectors by a hat (e.g., $\hat{x}$), matrices by bold upper-case letters (e.g., A), and scalars by plain lower-case letters (e.g., *t*). A rotation $R_{ab}$ denotes the rotation that maps coordinates from frame {a} to frame {b}; the translation of the origin of frame {a} expressed in frame {b} is denoted $p_{ab}$. Throughout the paper, we use feature to denote a tracked 2D keypoint, correspondence a 3D–2D match used for pose estimation, and landmark a triangulated 3D point maintained by the backend.

We denote camera frames by {c} and body/IMU frames by {b}. The camera–IMU extrinsic transform is fixed and written as $T_{bc}=\left[\;R_{bc}|p_{bc}\;\right]$, i.e., the relative pose between camera and IMU remains constant over time. IMU propagation between two time instants $t_{i}$ and $t_{j}$ follows the standard discrete integration of the IMU measurements. Let ${\tilde{\omega }}_{k}$ and ${\tilde{a}}_{k}$ be the raw gyroscope and accelerometer readings at time $t_{k}$, with corresponding biases $b_{k}^{g}$ and $b_{k}^{a}$, and let $\Delta t=t_{j}-t_{i}$ denote the sampling interval (for compactness we sometimes write Δt when sampling is uniform). The discrete IMU integration is expressed as:

(1) $$\begin{array}{cc} R_{j} & =R_{i}\prod_{k=i}^{j-1}\mathrm{Exp}\;\left(\left({\tilde{\omega }}_{k}-b_{k}^{g}-\eta_{k}^{gd}\right)\Delta t\right), \end{array}$$

(2) $$\begin{array}{cc} v_{j} & =v_{i}+g\;\Delta t_{ij}+\sum_{k=i}^{j-1}R_{k}\left({\tilde{a}}_{k}-b_{k}^{a}-\eta_{k}^{ad}\right)\Delta t, \end{array}$$

(3) $$\begin{array}{cc} p_{j} & =p_{i}+\sum_{k=i}^{j-1}\left[v_{k}\Delta t+\frac{1}{2}g\Delta t^{2}+\frac{1}{2}R_{k}\left({\tilde{a}}_{k}-b_{k}^{a}-\eta_{k}^{ad}\right)\Delta t^{2}\right]. \end{array}$$

where $R_{i}$, $R_{j}$ are the body rotations at $t_{i}$, $t_{j}$; $v_{i}$, $v_{j}$ are linear velocities; $p_{i}$, $p_{j}$ are positions; g is the gravity vector; and $\eta_{k}^{gd}$, $\eta_{k}^{ad}$ denote measurement noise terms for gyro and accelerometer, respectively. Exp(·) is the SO(3) exponential map.

We assume that, over the short time span used for initialization, sensor biases are approximately constant:

$$\begin{array}{cc} b_{k}^{g} & \approx b^{g},\;b_{k}^{a}\approx b^{a},\;\forall \;k\in \left[i,\ldots ,j\right] \end{array}$$

These notational conventions and assumptions are used throughout the initialization derivation and in the IMU–vision coupling that follows.

### 4.2. GNC Preliminaries

**Figure 3:**
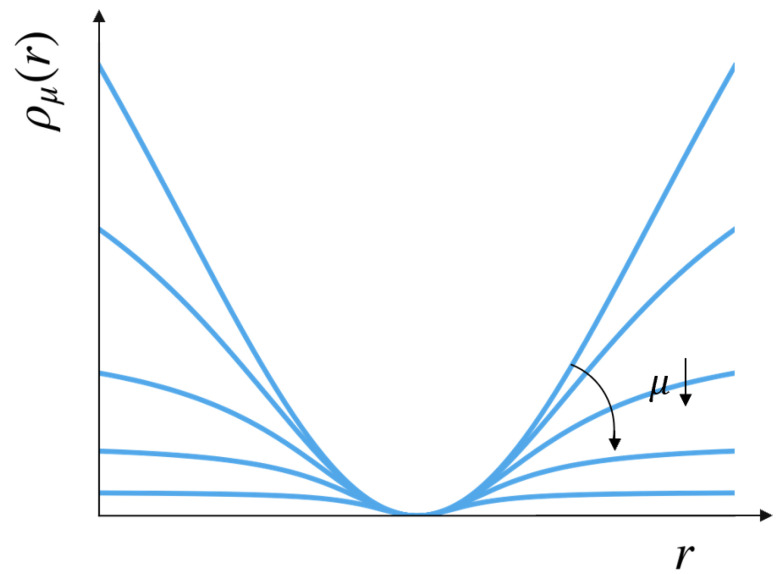
Illustration of the GNC surrogate penalty family. The continuation parameter μ controls a sequence of surrogate penalties $\rho_{\mu }\left(r\right)$ that gradually approach a stronger robust shape. For large μ, the surrogate is smoother and closer to a quadratic penalty; as μ decreases, the penalty becomes increasingly robust to large residuals by flattening its growth, thereby reducing the influence of incompatible measurements.

For completeness, we briefly summarize only the aspects of the graduated non-convexity (GNC) framework that are directly used in the proposed initialization and pose-estimation modules. Many robust estimation problems can be written as

(4) $$\min_{\theta }\sum_{i=1}^{N}\rho \;\left(r_{i}{\left(\theta \right)}^{2}\right),$$

where θ denotes the unknown variables, $r_{i}\left(\theta \right)$ is the residual associated with measurement *i*, and ρ(·) is a robust penalty used to suppress outliers. Directly optimizing (4) is often difficult because the desired robust loss is typically non-convex. Figure 3 illustrates the family of surrogate penalties used in GNC: the continuation parameter gradually deforms the objective from an easier and smoother shape toward a more strongly robust one.

Graduated non-convexity (GNC) addresses this difficulty by solving a sequence of surrogate problems controlled by a continuation parameter, starting from an easier objective and gradually approaching the target robust objective. In practice, this process can be interpreted as iterative reweighting,

(5) $$\min_{\theta }\sum_{i=1}^{N}w_{i}\;r_{i}{\left(\theta \right)}^{2},$$

where the weights $w_{i}\in \left[0,1\right]$ are updated according to the current residuals and the GNC continuation schedule. Measurements consistent with the current estimate retain large weights, while incompatible measurements are progressively downweighted. As μ decreases, the surrogate penalty becomes flatter for large residuals, which corresponds to assigning smaller effective weights to measurements that are increasingly inconsistent with the current estimate.

Following the general GNC framework in [27], we use GNC in this work as a practical robustification tool in two failure-critical stages of the pipeline, namely robust initialization and per-frame pose estimation, rather than as a new theoretical contribution.

### 4.3. Initialization with NEC and GNC-Based Gyroscope Bias Estimation

Visual–inertial initialization is brittle when feature tracking is degraded or the motion excitation is weak. We therefore estimate the gyroscope bias using a Normal Epipolar Constraint (NEC) formulation embedded in a Graduated Non-Convexity (GNC) framework, and guard initialization with a two-stage observability test that rejects windows with insufficient excitation or unstable geometry.

#### 4.3.1. GNC-Based Gyroscope Bias Estimation

We adopt the Normal Epipolar Constraint (NEC), originally introduced in [29], to improve robustness against outliers and degraded feature tracking. NEC encodes the epipolar-plane geometry induced by moving feature correspondences without requiring explicit essential-matrix decomposition.

**Figure 4:**
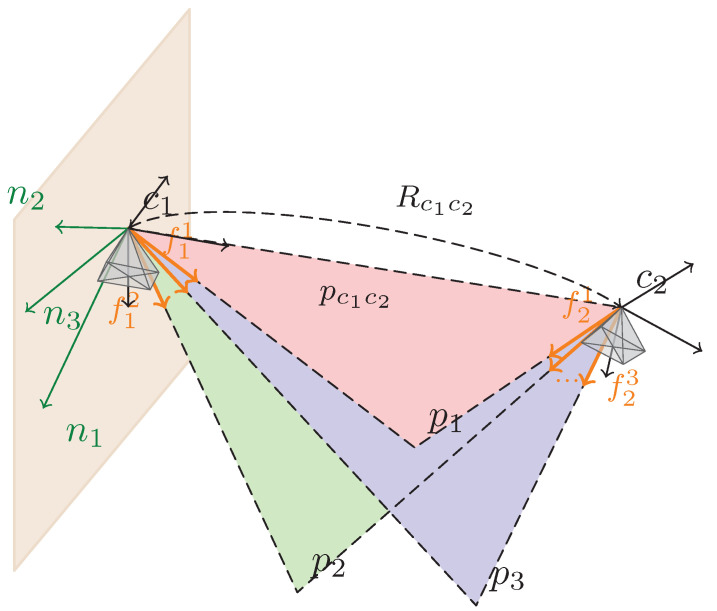
Epipolar plane geometry for the normal epipolar constraint (NEC). A matched feature observed in frames $\left(c_{1},c_{2}\right)$ defines two bearing rays ${\hat{f}}_{1}^{k}$ and ${\hat{f}}_{2}^{k}$; rotating ${\hat{f}}_{2}^{k}$ into $c_{1}$ yields an epipolar plane whose normal is $n^{k}\propto {\hat{f}}_{1}^{k}\times \left(R_{c_{1}c_{2}}{\hat{f}}_{2}^{k}\right)$. Inliers satisfy ${\left(n^{k}\right)}^{⊤}p_{c_{1}c_{2}}=0$ since the baseline $p_{c_{1}c_{2}}$ lies in the plane. Different colored shaded regions denote different epipolar planes; orange arrows denote bearing vectors, green arrows denote plane normals, and dashed lines indicate geometric connections used for visualization.

Let ${\hat{f}}_{i}^{k}$ and ${\hat{f}}_{j}^{k}$ denote the unit bearing vectors of correspondence *k* observed in camera frames $\left\{c_{i}\right\}$ and $\left\{c_{j}\right\}$, respectively. As illustrated in Figure 4, the two back-projected rays and the camera baseline lie on a common epipolar plane. The normal of this plane, expressed in frame $\left\{c_{i}\right\}$, is

(6) $$\begin{array}{c} n^{k}\propto {\hat{f}}_{i}^{k}\times \left(R_{c_{i}c_{j}}\;{\hat{f}}_{j}^{k}\right)={\left[{\hat{f}}_{i}^{k}\right]}_{\times }\;R_{c_{i}c_{j}}\;{\hat{f}}_{j}^{k}. \end{array}$$

Following [4], we use NEC residuals to refine the gyroscope bias by expressing the inter-frame rotation as a function of the bias through IMU preintegration. With the fixed camera–IMU extrinsic $T_{bc}=\left[\;R_{bc}|p_{bc}\;\right]$, the relative camera motion between frames can be written as

(7) $$\begin{array}{cc} R_{c_{i}c_{j}} & =R_{bc}^{⊤}R_{b_{i}b_{j}}R_{bc},\\ p_{c_{i}c_{j}} & =R_{bc}^{⊤}\left(p_{b_{i}b_{j}}+R_{b_{i}b_{j}}p_{bc}-p_{bc}\right). \end{array}$$

where $\left[\;R_{b_{i}b_{j}}|p_{b_{i}b_{j}}\;\right]$ is the IMU pre-integrated motion from $b_{i}$ to $b_{j}$.

To model the influence of gyroscope bias $b_{g}$, the pre-integrated rotation is linearized as

(8) $$\begin{array}{c} R_{b_{i}b_{j}}=\gamma_{b_{j}}^{\;b_{i}}\;\mathrm{Exp}\;\left(J_{b^{g}}^{\gamma_{b_{j}}^{\;b_{i}}}\;b^{g}\right) \end{array}$$

where $\gamma_{b_{j}}^{\;b_{i}}$ is the nominal pre-integrated rotation and $J_{b_{g}}^{\gamma_{b_{j}}^{\;b_{i}}}$ denotes the Jacobian of the pre-integration with respect to the gyroscope bias $b^{g}$. This Jacobian captures the sensitivity of the pre-integrated rotation to bias perturbations.

By substituting the corrected rotation into the NEC formulation, the objective function becomes:

(9) $$\min_{b^{g},{\hat{t}}_{ij}}\sum_{k}{\left[{\hat{f}}_{i}^{k}\cdot \left(R_{ij}\left(b^{g}\right){\hat{f}}_{j}^{k}\times {\hat{t}}_{ij}\right)\right]}^{2},$$

which follows the formulation in [4].

To suppress outliers, we embed NEC into a GNC framework [27]. For each correspondence *k*, define

(10) $$n_{k}\left(b^{g}\right)={\left[{\hat{f}}_{i}^{k}\right]}_{\times }R_{bc}^{⊤}\gamma_{b_{j}}^{\;b_{i}}\;\mathrm{Exp}\;\left(J_{b^{g}}^{\gamma_{b_{j}}^{\;b_{i}}}\;b^{g}\right)R_{bc}{\hat{f}}_{j}^{k}$$

and its normalized form

(11) $$\begin{array}{cc} m_{k}\left(b^{g}\right) & =\frac{n_{k}\left(b^{g}\right)}{\sqrt{∥n_{k}\left(b^{g}\right){∥}^{2}+\varepsilon }}. \end{array}$$

where ε prevents numerical issues near zero.

Given GNC weights $w_{k}\in \left[0,1\right]$, we accumulate the weighted second-moment matrix

(12) $$\begin{array}{cc} \tilde{M}\left(b^{g}\right) & =\sum_{k=1}^{N}w_{k}\left(m_{k}\left(b^{g}\right)\right){\left(m_{k}\left(b^{g}\right)\right)}^{⊤}, \end{array}$$

Let $\hat{p}$ denote the unit translation direction associated with $c_{i}\to c_{j}$. Under the weighted squared NEC residuals, $\hat{p}$ is obtained by

(13) $$\begin{array}{c} \min_{∥p∥=1}\sum_{k=1}^{N}w_{k}{\left(p^{T}m_{k}\left(b^{g}\right)\right)}^{2}\;=\;\min_{∥p∥=1}p^{T\;}\tilde{M}\left(b^{g}\right)\;p \end{array}$$

By the Rayleigh quotient theorem, the solution is the eigenvector corresponding to the smallest eigenvalue:

(14) $$\begin{array}{cc} p(b^{g}) & =\mathrm{eigmin}\;\left(\tilde{M}\left(b^{g}\right)\right). \end{array}$$

The resulting normalized projection residual is

(15) $$\begin{array}{cc} {\hat{r}}_{k}^{2}\left(b^{g}\right) & ={\left(m_{k}{\left(b^{g}\right)}^{⊤}p\left(b^{g}\right)\right)}^{2}\geq 0. \end{array}$$

Within GNC, sample weights are updated using the closed-form schedule of truncated least squares:

(16) $$\begin{array}{cc} w_{k}^{\left(t\right)} & =\left\{\begin{array}{cc} 0, & {\left({\hat{r}}_{k}^{\left(t\right)}\right)}^{2}\in \left[\frac{\mu +1}{\mu }{\bar{c}}^{2},\;+\infty \right],\\ \frac{\bar{c}}{{\hat{r}}_{k}^{\left(t\right)}}\sqrt{\mu (\mu +1)}-\mu , & {\left({\hat{r}}_{k}^{\left(t\right)}\right)}^{2}\in \left[\frac{\mu }{\mu +1}{\bar{c}}^{2},\;\frac{\mu +1}{\mu }{\bar{c}}^{2}\right],\\ 1, & {\left({\hat{r}}_{k}^{\left(t\right)}\right)}^{2}\in \left[0,\;\frac{\mu }{\mu +1}{\bar{c}}^{2}\right]. \end{array}\right. \end{array}$$

where $\bar{c}$ is the noise bound and μ≥1 controls the degree of non-convexity. The inner loop terminates when the objective (or $\sum_{k}w_{k}$) changes only marginally; otherwise, μ is increased (e.g., μ←αμ with α=1.4), and the procedure repeats until convergence or a maximum number of iterations is reached.

#### 4.3.2. Two-Stage Observability Test

Premature initialization typically stems from (i) insufficient excitation (bias–gravity ambiguity) or (ii) numerical instability caused by near-degenerate geometry. We therefore use a two-stage gate: Stage I checks rotational excitation, and Stage II verifies spectral stability of the linearized system.

#### Stage I—Rotational Excitation

For each adjacent camera-frame pair $\left(c_{i},c_{i+1}\right)$, we obtain the IMU-predicted relative camera rotation from IMU preintegration and the fixed camera–IMU extrinsic, and denote it by $R_{c_{i}c_{i+1}}$ (cf. Equation (7)). For a feature *k* with unit bearing ${\hat{f}}_{i}^{\;k}$ observed in frame $c_{i}$, the IMU-rotation-compensated prediction of its bearing in the next frame is

(17) $${\hat{f}}_{i+1}^{k,\mathrm{pred}}=R_{c_{i}c_{i+1}}{\hat{f}}_{i}^{k}.$$

The angular disparity between the predicted bearing and the actual measurement is defined by

(18) $$\begin{array}{cc} \theta_{i}^{k} & =atan2\;\left(\;∥{\hat{f}}_{i+1}^{k,\mathrm{pred}}\times {\hat{f}}_{i+1}^{k}∥,\;{\hat{f}}_{i+1}^{k,\mathrm{pred}}\cdot {\hat{f}}_{i+1}^{k}\;\right). \end{array}$$

Over the time span $\Delta t^{k}$ during which feature *k* is continuously tracked, the mean rotation-compensated angular-disparity rate is computed as

(19) $$\begin{array}{c} {\bar{\omega }}^{k}\;=\;\frac{1}{\Delta t^{k}}\sum_{i}\theta_{i}^{k} \end{array}$$

Stage I accepts the window when at least 50 tracked features satisfy ${\bar{\omega }}^{k}>\omega_{\mathrm{th}}$, indicating sufficient motion excitation for bias observability. This signals adequate rotational excitation in the visual measurements after compensating for the IMU-predicted rotation.

#### Stage II—Spectral Stability of LiGT

If Stage I is satisfied, we build the linearized LiGT system following the linear global translation (LiGT) formulation in [30] (depths are analytically marginalized), and form the Hessian

(20) $$\begin{array}{cc} H_{m} & =A_{m}^{\;T}A_{m}. \end{array}$$

We use the smallest eigenvalue of $H_{m}$ as a scalar spectral measure,

(21) $$\begin{array}{c} \lambda_{m}\;=\;\lambda_{\min}\;\left(H_{m}\right), \end{array}$$

and monitor its relative variation between consecutive constructions. In our implementation, $H_{m}$ is re-constructed once per window update as a new frame enters the candidate initialization window. The relative spectral variation is defined as

(22) $$\begin{array}{cc} \delta_{m}\; & =\frac{\left|\lambda_{m}-\lambda_{m-1}\right|}{\lambda_{m-1}+\epsilon_{\lambda }}, \end{array}$$

where $\epsilon_{\lambda }$ is a small constant to avoid numerical issues when $\lambda_{m-1}$ is close to zero.

Stage II accepts the window for initialization only when the spectral metric remains stable across successive constructions, i.e., $\delta_{m}<\delta_{\mathrm{th}}$ for *K* consecutive evaluations. We use $\delta_{\mathrm{th}}=0.25$ and K=2−4 in all experiments. This gate ensures that incorporating new measurements does not drastically change the conditioning of the linear system, thereby reducing the risk of numerically unstable initialization.

Stage I checks for sufficient rotational excitation using the rotation-compensated angular-disparity rate threshold $\omega_{\mathrm{th}}$. Stage II assesses numerical identifiability of translation- and acceleration-related parameters by monitoring the spectral stability of $H_{m}$.

Notably, the LiGT constraint in [30] is robust under common degenerate motions, including local pure rotations and near-collinear trajectories. This robustness stems from its global aggregation across multiple frames and analytical depth marginalization.

### 4.4. Hierarchical Outlier Rejection for Robust Tracking

Dynamic environments introduce feature noise, depth uncertainty, and spatial clustering, which jointly degrade pose estimation. We design a lightweight hierarchical outlier-rejection pipeline that couples an INS-aided frontend with a GNC-enhanced efficient perspective-n-point (EPnP) solver, temporal landmark filtering, and spatial voxelization.

#### 4.4.1. Frontend and Keyframe Management

The frontend follows an INS-aided optical-flow strategy. Images are partitioned into fixed grids (e.g., 200×200 pixels) to enforce spatially uniform feature coverage. For landmarks without depth, INS-predicted feature positions are used to initialize optical flow; for landmarks with established depth, 3D positions are projected using the INS-predicted camera pose to guide the search window.

Keyframes are selected using rotation-compensated average disparity: a new keyframe is created when the disparity exceeds 15 pixels, and a keyframe is force-inserted every 0.15 s to avoid backend starvation. Removing keyframes uses a straightforward marginalization of their states and associated landmarks from the factor graph.

#### 4.4.2. GNC-EPnP: Graduated Non-Convex Pose Estimation

Here GNC is used as a robustification mechanism rather than introduced as a new estimation theory; the contribution lies in adapting it to the EPnP-based pose-estimation stage within a real-time VIO pipeline.

Standard efficient perspective-n-point (EPnP) [31] is efficient but sensitive to outliers due to its least-squares nature. We embed EPnP into GNC to obtain continuous, hypothesis-free outlier suppression.

Each 3D landmark $p_{w}^{i}$ is represented as a linear combination of four virtual control points ${\left\{c_{w}^{j}\right\}}_{j=1}^{4}$:

(23) $$p_{w}^{i}=\sum_{j=1}^{4}\alpha_{ij}\;c_{w}^{j}.$$

The same holds in the camera frame:

(24) $$p_{c}^{i}=\sum_{j=1}^{4}\alpha_{ij}\;c_{c}^{j}.$$

Using camera intrinsics K and image observations $u_{j}$, the projection constraints produce a homogeneous linear system

(25) Mx=0,

where $x={\left[c_{c}^{1⊤},c_{c}^{2⊤},c_{c}^{3⊤},c_{c}^{4⊤}\right]}^{⊤}$ collects control points in the camera frame and M is assembled from per-observation coefficients.

For observation *i*, let $M_{i}\in R^{2\times 12}$ be its two-row block and precompute

(26) $$\begin{array}{c} H_{i}=M_{i}^{⊤}M_{i},\;r_{i}^{2}=x^{⊤}H_{i}x. \end{array}$$

Introducing weights $w_{i}\in \left[0,1\right]$, the weighted normal matrix is

(27) $$\begin{array}{cc} H_{w} & =\sum_{i=1}^{N}w_{i}\;H_{i} \end{array}$$

Within GNC, sample weights are updated using the closed-form truncated least-squares (TLS) schedule (cf. (16)), and the solution for x is obtained via eigendecomposition of $H_{w}$. With precomputed $H_{i}$, each iteration remains O(n), preserving the efficiency of EPnP while improving robustness.The detailed iterative procedure is summarized in Algorithm 1.

#### 4.4.3. Temporal Landmark Classification

Let $\tau_{i}$ denote the tracking duration (number of frames) and $\nu_{i}$ the number of factor graph optimization (FGO) refinements for landmark *i*. Partition the landmark database L into long-term and short-term subsets:

(28) $$\begin{array}{cc} L_{\mathrm{long}} & =\{\;i\in L|\tau_{i}\geq \tau_{\mathrm{th}}\;\mathrm{and}\;\nu_{i}\geq \nu_{\mathrm{th}}\},\\ L_{\mathrm{short}} & =L\setminus L_{\mathrm{long}}. \end{array}$$

Typical thresholds are $\tau_{\mathrm{th}}=5$ frames and $\nu_{\mathrm{th}}=3$ optimizations. Long-term landmarks have gone through multiple triangulation/FGO cycles and thus possess higher-confidence depths.

#### 4.4.4. Spatial Voxelization and IMU-Aided Short-Term Integration

Voxelization for uniform control-point selection. To obtain a spatially well-distributed set of control points for EPnP, we voxelize the long-term landmarks in 3D using a grid of size $\delta_{v}$ (e.g., 0.5 m). From each non-empty voxel $V_{j}$, we retain a single representative landmark (the closest one to the camera), forming

(29) $$L_{\mathrm{vox}}=\left\{\;\arg\min_{l\in V_{j}}{∥p_{l}^{c}∥}_{2}\;|\;V_{j}\neq \emptyset \right\}.$$

Importantly, $L_{\mathrm{vox}}$ is used only to compute EPnP control points and is not included as correspondences in the subsequent GNC-EPnP optimization.
**Algorithm 1** GNC-EPnP Inlier Solver**Require:** 
${\left\{p_{i}\right\}}_{i=1}^{N},{\left\{u_{i}\right\}}_{i=1}^{N},{\left\{w_{i}^{\mathrm{long}}\right\}}_{i=1}^{N},\left(R_{\mathrm{init}},t_{\mathrm{init}}\right),{\bar{\sigma }}^{2},\gamma ,N_{\max},\epsilon$**Ensure:** 
Pose $\left(R^{*},t^{*}\right)$, inlier set I, flags (early_exit,stopped_by_tol)1:Keep indices $J=\{i|w_{i}^{\mathrm{long}}>0\}$2:Build M from downsampled points and barycentric weights3:Initialize $\left(R,t\right)\leftarrow \left(R_{\mathrm{init}},t_{\mathrm{init}}\right)$4:early_exit←false, stopped_by_tol←false5:Compute squared residuals $r_{i}^{2}$ and $\mathrm{den}=\frac{2\max_{i}r_{i}^{2}}{{\bar{\sigma }}^{2}}-1$6:**if** den<0 **then**7:    I={1,2,…,N}8:    $\left(R^{*},t^{*}\right)\leftarrow \left(R,t\right)$9:    early_exit←true **return** $\left(R^{*},t^{*}\right),I,\left(\mathrm{early}\_\mathrm{exit},\mathrm{stopped}\_\mathrm{by}\_\mathrm{tol}\right)$10:**else**11:    $\mu \leftarrow \mathrm{clip}(1/\mathrm{den},0,\mu_{\max})$12:**end if**13:Initialize weights $w_{i}$, set $W_{i}\leftarrow w_{i}\cdot w_{i}^{\mathrm{long}}$14:μ←μ·γ, ${\mathrm{cost}}_{\mathrm{prev}}\leftarrow \infty$15:**for** *t* = 1 **to** $N_{\max}$ **do**16:    Solve weighted EPnP with *W* and M to update (R,t)17:    Update residuals $r_{i}^{2}$, thresholds, and weights $W_{i}\leftarrow w_{i}\cdot w_{i}^{\mathrm{long}}$18:    Compute normalized cost $\bar{C}=\frac{\sum_{i}W_{i}r_{i}^{2}}{\sum_{i}W_{i}}$19:    **if** $|\bar{C}-{\mathrm{cost}}_{\mathrm{prev}}|<\epsilon$ **then**20:        $I=\{i|w_{i}\geq w_{\mathrm{thr}}\}$21:        $\left(R^{*},t^{*}\right)\leftarrow \left(R,t\right)$22:        stopped_by_tol←true **return** $\left(R^{*},t^{*}\right),I,\left(\mathrm{early}\_\mathrm{exit},\mathrm{stopped}\_\mathrm{by}\_\mathrm{tol}\right)$23:    **end if**24:    μ←μ·γ, ${\mathrm{cost}}_{\mathrm{prev}}\leftarrow \bar{C}$25:**end for**26:$I=\{i|w_{i}\geq w_{\mathrm{thr}}\}$27:$\left(R^{*},t^{*}\right)\leftarrow \left(R,t\right)$ **return** $\left(R^{*},t^{*}\right),I,\left(\mathrm{early}\_\mathrm{exit},\mathrm{stopped}\_\mathrm{by}\_\mathrm{tol}\right)$

IMU-prior initialization for EPnP correspondences. Let $L_{\mathrm{EPnP}}=\left\{\;l|N_{l}>3\;\right\}$ denote the landmarks eligible for EPnP, i.e., those observed in more than three frames. For each $l\in L_{\mathrm{EPnP}}$, we initialize a prior weight using the IMU-predicted motion. Given the IMU-predicted pose $\left(R_{init},\;t_{init}\right)$, we compute the prior EPnP algebraic residual $r_{\mathrm{IMU}}$ and map it to an initial weight $w_{l}^{\mathrm{prior}}$ (cf. (16)):

(30) $$r_{\mathrm{IMU}}^{2}={∥M\;x^{\mathrm{pred}}∥}_{2}^{2},\;c_{c}^{j,\mathrm{pred}}=R_{init}c_{w}^{j}+t_{init},\;j=1,\ldots ,4$$

where $x={\left[c_{w}^{1⊤},\;c_{w}^{2⊤},\;c_{w}^{3⊤},\;c_{w}^{4⊤}\right]}^{⊤}$ stacks the control points expressed in the first camera frame (treated as the world frame). To avoid injecting highly inconsistent measurements, we optionally discard correspondences with small prior weights, i.e., $w_{l}^{\mathrm{prior}}\leq w_{\mathrm{th}}$, where $w_{\mathrm{th}}=0.2$. Accordingly, the final correspondence set used by GNC-EPnP is

(31) $$L_{\mathrm{GNC}}=\left\{\;l\in L_{\mathrm{EPnP}}|w_{l}^{\mathrm{prior}}>w_{\mathrm{th}}\;\right\}.$$

Overall, temporal screening improves depth reliability by prioritizing sufficiently tracked and refined landmarks (Equation (28)). Spatial voxelization enhances EPnP conditioning by suppressing clustered features (Equation (29)). IMU-aided weighting provides soft initialization for GNC iterations (Equation (30)), accelerating convergence in high-outlier regimes.

## 5. Experiments

### 5.1. Experimental Setup and Evaluation Protocol

Experiments are conducted on a desktop PC with an Intel Core i9-12700 CPU and 48 GB RAM (no GPU). The system is evaluated in real-time replay under a multi-threaded implementation: tracking, mode selection, and backend optimization run in three dedicated threads; feature extraction within tracking is parallelized by image partitioning; and the GNC weight update inside a 10-frame window is parallelized across 9 threads.

We evaluate the proposed method on all 11 sequences of EuRoC MAV [32] and all sequences of VIODE [33]. The two datasets play complementary roles in the evaluation. EuRoC is mainly used for controlled system-level benchmarking, initialization analysis, and ablation studies, whereas VIODE is used primarily to assess robustness under degraded visual conditions and stronger dynamic interference. Accordingly, the compared baselines are selected based on the objective of each experimental block and fairness considerations, rather than being enforced uniformly across all settings. VINS-Fusion serves as the most direct implementation-controlled baseline in initialization-related and module-focused comparisons. To complement it with a more recent and widely used open-source VIO baseline, we additionally include OpenVINS [34] in the EuRoC system-level comparison. OpenVINS represents a standard tightly coupled visual–inertial pipeline with a public and reproducible implementation and has been widely adopted in recent comparative evaluations. In the dynamic-scene evaluation on VIODE, we further include ORB-SLAM3 as a strong system-level reference. We do not treat ORB-SLAM3 as a directly matched baseline in all EuRoC VIO-focused comparisons, since its accuracy can benefit substantially from loop closing and global optimization, whereas the VIO baselines considered here are evaluated without loop closure.

Accuracy is measured mainly by the root-mean-square error (RMSE) of the absolute trajectory error (ATE) after SE(3) alignment to ground truth under a consistent protocol. For the main EuRoC system-level comparison, we additionally report relative trajectory error (RTE) over fixed segment lengths to characterize local drift behavior. To avoid conflating the pre-initialization transient with steady-state tracking quality, ATE is computed only after initialization is completed. Each EuRoC sequence is replayed five times, and we report the mean RMSE. For compact presentation, the main result tables report mean values; For compact presentation, the main result tables report mean values; in addition, the run-to-run standard deviation of RISE-VIO (full) is reported together with the corresponding EuRoC comparison results as a representative measure of stochastic variation. Empirically, RISE-VIO (a) and (b) exhibit a similar level of run-to-run variation under the same sequences and replay settings.

Runtime is measured as wall-clock compute time using a high-resolution clock. When reporting algorithmic runtime, we exclude dataset I/O and trajectory dumping/logging to ensure stable and fair comparisons across platforms and storage backends. In dynamic-scene evaluation, “*” denotes tracking failure or catastrophic divergence, i.e., no valid trajectory is produced for evaluation under the same alignment protocol. The start of evaluation is defined as the first timestamp at which the estimator switches to normal tracking after the initialization flag is set.

The current evaluation is conducted through controlled replay of public benchmark datasets, providing a reproducible basis for examining initialization behavior, visual robustness, and trajectory accuracy under a unified protocol. This setup is well suited for controlled comparison, but it does not fully reflect several factors that can affect real-world performance, such as camera–IMU calibration quality, time synchronization, onboard computational constraints, and prolonged exposure to severe illumination changes or dynamic interference. Accordingly, while the proposed method is developed within a standard visual–inertial framework and does not rely on offline training or scene-specific priors, further validation under fully online deployment conditions remains an important direction for future work.

**Implementation details.** The main implementation used in this work was developed primarily on top of IC-GVINS, with the GPS-related components removed for the present study. Since IC-GVINS itself inherits design elements from VINS-Fusion, the resulting pipeline remains structurally similar to VINS-Fusion. In addition, because different experiments are designed to answer different questions—module-level initialization analysis, controlled PnP robustness evaluation, and end-to-end system comparison—the exact set of baselines is not identical across all subsections. Where possible, we use implementation-controlled comparisons within the same backbone; for full-system benchmarking, we additionally include representative external baselines. Unless otherwise stated, a fixed set of hyperparameters is used in all experiments: $\varepsilon =10^{-12}$ for normalization, $\bar{c}=0.05$ for the TLS-GNC schedule, and $\omega_{\mathrm{th}}=0.15\;\mathrm{rad}/s$ with a minimum of 50 features for the Stage I excitation test.

### 5.2. Robust Initialization on EuRoC

We first isolate the effect of initialization by integrating different initialization strategies into the same VINS-Fusion backbone while keeping the remaining frontend/backend settings unchanged. We compare vanilla VINS-Fusion with a DRT-style initializer and the proposed GNC-DRT-INIT, under two feature budgets. Results are summarized in Table 1.

Overall, robust initialization yields the largest gains on sequences where early mismatches, degraded tracking, or weak excitation make bias–gravity separation brittle (e.g., the MH/V2 difficult sequences). On easier sequences, the gain becomes marginal and may occasionally fluctuate, consistent with a typical robustness trade-off: downweighting inconsistent measurements improves worst-case stability, but can slightly reduce information usage when the data are already clean. The two feature budgets (150 vs. 200 tracks) further expose this practical trade-off. Tracking more points often increases the chance of admitting low-quality correspondences and mismatches, especially in the early stage when motion is limited and the tracker is not yet stabilized. Under this noisier setting, gnc_drt_init tends to show a clearer advantage over the drt_init baseline. At the same time, using a larger tracking budget can introduce more outliers later in the sequence, which may occasionally offset part of the benefit and lead to slightly higher RMSE on some runs. Even in those cases, Table 1 suggests that gnc_drt_init remains comparable to, and often better than, drt_init. This motivates the system-level ablation in Section 5.5, where we separate “startup robustness” from “running-time robustness”.

**Figure 5:**
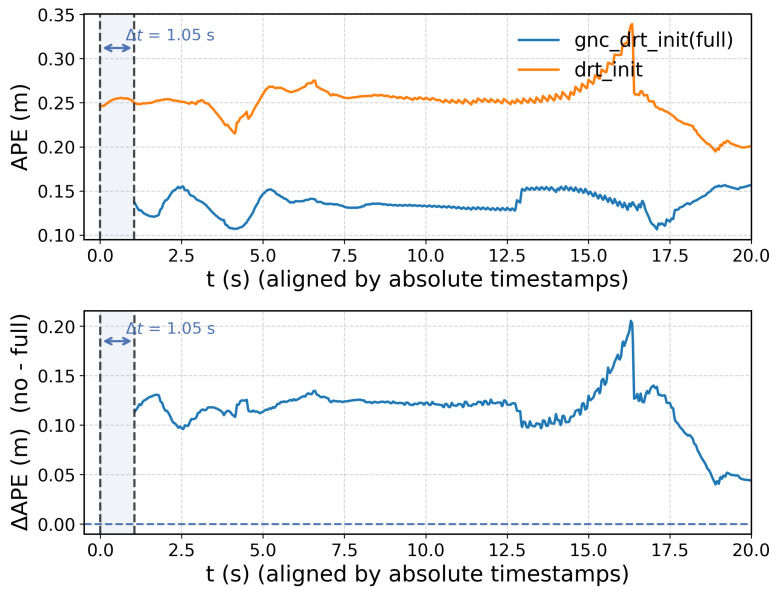
Effect of the two-stage observability gate in GNC-DRT-INIT (full) on initialization timing and early accuracy. Results are shown on MH_05_difficult. Absolute-position error (APE) over the first 20 s of the sequence (**top**) for the same RISE-VIO pipeline instantiated with two initialization strategies: gnc_drt_init(full) (with the proposed two-stage observability gate) and drt_init (without the gate). The (**bottom**) plot shows the difference $\Delta \mathrm{APE}={\mathrm{APE}}_{\mathrm{drt}\_\mathrm{init}}-{\mathrm{APE}}_{\mathrm{gnc}\_\mathrm{drt}\_\mathrm{init}(\mathrm{full})}$, where positive values indicate lower error with gnc_drt_init(full). Curves are aligned by absolute timestamps to reveal the delayed activation induced by the gate (marked by Δt).

To further analyze the role of the proposed two-stage observability gate, Figure 5 provides a fine-grained comparison of early-stage behavior on the challenging MH_05_difficult sequence. Specifically, we visualize the absolute position error (APE) during the first 20 s for gnc_drt_init(full), which activates only after passing both observability checks, and for drt_init, which initializes immediately without gating.

As shown in the top plot, the two-stage gate introduces a short activation delay of approximately Δt=1.05 s, during which the system continues pure IMU propagation while accumulating informative motion and reliable visual tracks. Despite this slight postponement, gnc_drt_init(full) consistently achieves significantly lower early-stage APE than drt_init. The bottom plot further highlights this improvement through the error difference ΔAPE, which remains strictly positive over most of the interval, indicating a persistent accuracy gain. Notably, the gap grows rapidly immediately after initialization, reaching up to ∼0.20 m, and remains substantial throughout the first 20 s.

This behavior confirms that delayed activation is not intended to merely postpone initialization, but rather to select a more informative and numerically stable moment. By enforcing sufficient motion excitation (Stage I) and spectral conditioning (Stage II), the gate avoids premature bias–gravity separation under weak parallax or ill-conditioned geometry, thereby producing more reliable bootstrap states. As a result, the system enters the tightly coupled optimization phase with a significantly improved initial estimate, which translates into lower transient error and faster convergence. Importantly, the observed delay is short and does not introduce noticeable startup latency in typical sequences, while yielding a clear gain in robustness and early-stage accuracy.

### 5.3. Controlled Monte Carlo Perspective-n-Point (PnP) Evaluation

To quantify robustness under controlled outlier ratios, we conduct a Monte Carlo simulation with a virtual camera. In each trial, 3D–2D correspondences are generated via frustum sampling under fixed intrinsics, and outliers are injected by randomly replacing a subset of 2D observations within the image bounds while enforcing a minimum displacement from the original projection. The outlier ratio is swept from low to severe, and *T* trials are performed for each ratio (T=100 in all experiments).

**Figure 6:**
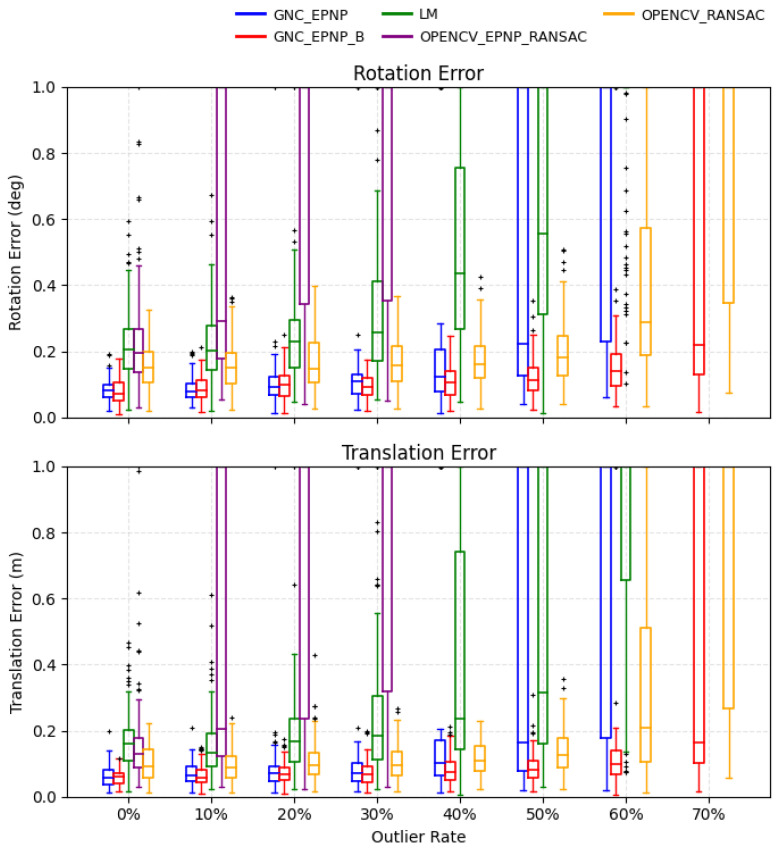
Monte Carlo PnP evaluation—rotation/translation errors. Rotation and translation error distributions (deg/m) across outlier ratios. For readability, the displayed y-range of both panels is limited to 1.0. In heavily degenerate cases where more than 80% of trials fall outside the displayed range, the corresponding boxplots are omitted because the central distribution is no longer informative within the plotted scale. The “+” symbols denote singular or degenerate trials, typically caused by severe outlier contamination.

**Figure 7:**
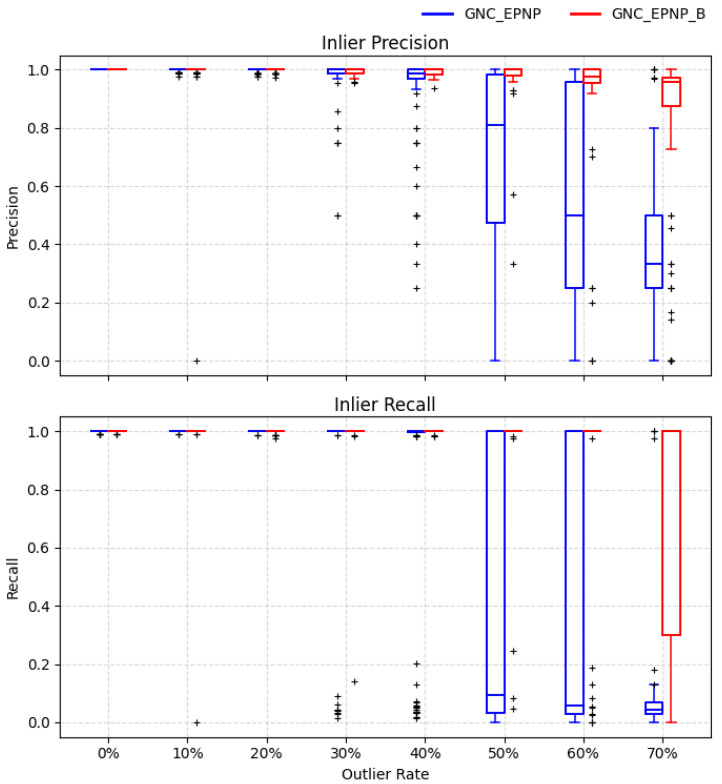
Monte Carlo PnP evaluation—inlier classification. Inlier classification measured by precision and recall across outlier ratios. In heavily degenerate cases where more than 80% of trials collapse to near-zero classification performance, the corresponding boxplots are omitted for visual clarity. The “+” symbols denote singular or degenerate trials, typically caused by severe outlier contamination.

We compare several representative robust PnP baselines, including OpenCV RANSAC-based iterative and EPnP variants, as well as an LM refinement baseline, against the proposed GNC-EPnP and its re-initialized variant GNC-EPnP-B. The results are shown in Figure 6 and Figure 7, which report the rotation/translation error distributions and the inlier classification behavior, respectively.

The results reveal different advantages in different outlier regimes. In the low-outlier regime, GNC-EPnP achieves higher pose accuracy than the non-EPnP baselines, indicating that its weighted formulation preserves the efficiency of EPnP and quality. As the outlier ratio increases, its advantage becomes more pronounced relative to OpenCV-EPnP-RANSAC, whose performance degrades rapidly once the effective inlier fraction falls below what the sampling budget can reliably support. By contrast, the graduated reweighting scheme progressively suppresses large-residual correspondences, allowing GNC-EPnP to maintain more stable estimation under heavier contamination.

The re-initialized variant GNC-EPnP-B further improves this behavior. Compared with the strongest OpenCV-RANSAC baseline, it better combines the high accuracy observed in the low-outlier regime with the robustness retained in the high-outlier regime. This improvement is particularly evident in the extreme case where the initial solution is supported by very few inliers: the re-initialization provides a more reliable starting point for subsequent GNC reweighting and reduces the chance of remaining trapped near degenerate solutions.

### 5.4. Dynamic Scene Stress Test on VIODE

We evaluate robustness in dynamic environments on VIODE under four levels of moving-object interference (none/low/mid/high). Results are summarized in Table 2, where RISE-VIO corresponds to RISE-VIO (full).

Dynamic interference increases the outlier ratio and corrupts geometric constraints, which may induce severe drift or catastrophic divergence for some baselines (marked by “*”). The full robust pipeline remains stable and reduces ATE as the dynamic level increases, indicating that correspondence-level robustness becomes increasingly critical when a larger fraction of tracked features originate from moving objects.

**Figure 8:**
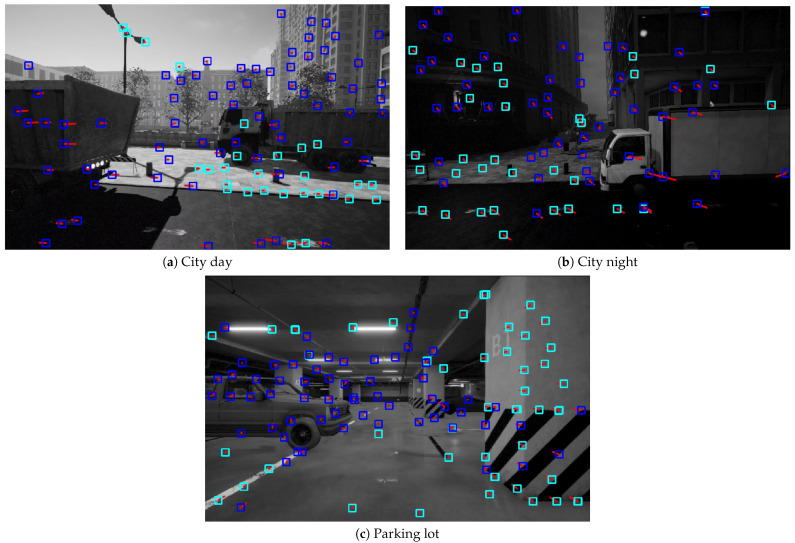
Qualitative visualization of feature/landmark status under dynamic interference on VIODE. (**a**) City day. (**b**) City night. (**c**) Parking lot. Light cyan boxes denote triangulated 3D landmarks that have been promoted to map points and are already included in backend optimization. Dark blue boxes indicate tracked 2D features that are still in the short-term track set (waiting for sufficient parallax/triangulation) and are therefore not yet optimized as landmarks.Red lines indicate the motion trajectories of corresponding points between consecutive frames.

Figure 8 provides a qualitative visualization of the online feature/landmark states during VIODE runs. Light-cyan boxes mark triangulated 3D landmarks that have already been inserted into backend optimization, whereas dark-blue boxes denote tracked 2D features that are maintained for short-term tracking but have not yet met the triangulation criteria (and thus are not optimized as landmarks).

It should be emphasized that the proposed GNC mechanism is intended to reject unreliable visual constraints rather than to compensate for the complete absence of visual information. In practice, the GNC-EPnP module is activated only when a sufficient number of valid long-track correspondences are available; otherwise, the visual update is skipped and the estimator continues with IMU-driven propagation and 2D feature tracking. Therefore, the system does not become numerically stuck merely because many visual weights become small or zero. However, under prolonged visual degradation, the estimator necessarily becomes more inertial-dominated and may accumulate drift, which reflects a general limitation of visual–inertial estimation rather than a failure mode specific to the proposed method.

### 5.5. System-Level Benchmarking and Ablation on EuRoC

We further benchmark the full system on EuRoC and perform ablation to isolate the contribution of each component. Table 3 compares VINS-Fusion, OpenVINS, RISE-VIO (a) (robust initialization only), RISE-VIO (b) (robust per-frame outlier rejection only), and RISE-VIO (full) (both enabled). Here, VINS-Fusion serves as the closest implementation-controlled baseline, while OpenVINS is included as a more recent representative open-source VIO system to make the comparison more comprehensive.

The results show clear complementarity between the two proposed modules. Robust initialization mainly improves early-stage stability and reduces the risk of biased initial states on sequences with difficult startup, whereas robust per-frame rejection improves resilience to intermittent tracking degradation and visual outliers during normal operation. The OpenVINS results also exhibit noticeable sequence-dependent variation, which is consistent with the sensitivity of VIO systems to initialization, front-end tracking behavior, and motion characteristics. Nevertheless, enabling both proposed modules yields the strongest overall performance, and RISE-VIO (full) achieves the best RMSE on most sequences. Moreover, the run-to-run variation of RISE-VIO (full) (Table 4) is small compared with the improvements obtained on the more challenging sequences, indicating that the observed gains are not simply caused by random fluctuation.

To further characterize local drift behavior beyond the global ATE metric, Table 5 additionally reports the relative trajectory error (RTE) over fixed segment lengths. In this part, we focus on VINS-Fusion and RISE-VIO variants, for which the comparison is most directly aligned with the implementation-level analysis. The RTE results are broadly consistent with the ATE comparison and provide a more local view of trajectory consistency in VIO. In particular, at shorter segment lengths, RISE-VIO (full) shows lower local drift on most sequences, while for longer segment lengths the relative advantage becomes more sequence-dependent.

### 5.6. Runtime and Real-Time Performance

We evaluate runtime under ROSbag replay using simulated time on the desktop PC described in Section 5.1. System-level cost is characterized by wall-clock runtime, CPU utilization, and peak resident memory. The wall-clock time is measured at the process level and thus includes a small constant overhead from launch/teardown and command invocation, which can make it slightly longer than the effective replay duration.

To characterize end-to-end responsiveness, we measure the delay between the timestamp carried by the odometry message header (nav_msgs/Odometry.header.stamp) and the current ROS time. Importantly, the header timestamp is set to the estimated state time (data time) rather than the publish time; this makes the reported delay reflect end-to-end latency through the processing pipeline. We summarize the delay by its mean, median, and 95th percentile to capture both typical latency and jitter.

Table 6 shows that both the benchmark and demo configurations sustain real-time replay on EuRoC. While the demo setup increases compute load (252% CPU and 798 MB peak RSS), it maintains bounded odometry latency (median ≈ 0.088 s and 95th percentile ≈ 0.142 s), indicating that the multi-threaded pipeline remains responsive under heavier execution. Here, CPU utilization is reported as the aggregated process-level usage across all CPU cores (i.e., values above 100% indicate multi-core parallel execution): 252% therefore corresponds to roughly 2.52 cores fully utilized. This number should be interpreted as evidence of effective parallelism in the tracking, mode-selection, and backend optimization threads rather than as single-core overload or a failure of real-time operation.

## 6. Conclusions

We presented RISE-VIO, a real-time INS-centric visual–inertial odometry system that improves robustness under degraded visual conditions through targeted GNC-based robustification of two failure-critical stages: initialization and per-frame pose estimation. The proposed GNC-DRT-INIT, combined with a two-stage observability gate, mitigates brittle bias–gravity separation under weak excitation and disturbed tracking, yielding consistent accuracy gains on challenging EuRoC sequences (Table 1 and Table 3). The IMU-prior-guided GNC-EPnP module further improves tolerance to dynamic mismatches, maintaining stable pose estimates under severe outlier contamination in the Monte Carlo PnP evaluation (Section 5.3) and reducing ATE in dynamic scenes (Table 2).

A key strength of RISE-VIO is that it improves robustness by strengthening two failure-critical estimation stages without introducing a heavy robust backend. This targeted integration preserves the efficiency and modularity of a standard real-time INS-centric visual–inertial odometry pipeline while improving resilience under weak texture, illumination variation, and high-outlier conditions. At the same time, the method still has several limitations. We observe an inherent robustness–efficiency trade-off: when correspondences are already clean, robust reweighting can slightly reduce nominal accuracy, and performance remains sensitive to IMU preintegration quality and time synchronization. Moreover, correspondence-level rejection may confuse near-field parallax with true dynamics in densely moving scenes, suggesting the need for richer motion cues beyond reprojection residuals. Under prolonged visual degradation or persistently weak excitation, the system may still fall back to inertial-dominated propagation and thus suffer from drift accumulation or delayed initialization.

From a deployment perspective, the proposed design is attractive because it preserves the overall structure and efficiency of a standard real-time INS-centric VIO pipeline, making it easier to integrate into existing systems than approaches that require full-backend reformulation. Nevertheless, practical use on real platforms still requires careful handling of calibration quality, sensor synchronization, abrupt illumination transitions, and long periods of weak visual observability. Future work will therefore focus on adaptive hyperparameter scheduling (e.g., scene-aware $\bar{c}$ and threshold selection), integrating lightweight semantic or motion cues to better disambiguate dynamics, extending the current targeted robustification to additional modules such as local bundle adjustment while preserving real-time performance, and validating the framework in broader real-world experiments beyond dataset playback. We also plan to further compare the proposed framework against more recent visual–inertial odometry backbones and investigate how the proposed modules can be more tightly integrated with such systems.

## Figures and Tables

**Table 1:** RMSE trajectory error comparison on EuRoC MAV. Here, raw denotes the baseline VINS-Fusion configuration (default 150 tracked features). We report results for VINS-Fusion with two initialization variants (drt_init and gnc_drt_init), each evaluated with 150/200 tracked features. Bold values indicate the best result(s) in each row.

Seq.	Raw	Drt150	Gnc150	Drt200	Gnc200
MH_01_easy	0.180	**0.151**	0.153	0.193	0.194
MH_02_easy	0.105	**0.086**	**0.086**	0.091	0.092
MH_03_medium	0.143	0.115	0.116	0.107	**0.100**
MH_04_difficult	0.216	0.205	0.199	0.208	**0.181**
MH_05_difficult	0.290	0.254	0.254	0.218	**0.183**
V1_01_easy	0.071	0.100	0.072	**0.069**	0.070
V1_02_medium	0.128	**0.093**	**0.093**	0.097	0.098
V1_03_difficult	0.170	0.163	0.163	**0.155**	**0.155**
V2_01_easy	0.142	0.080	0.070	0.065	**0.063**
V2_02_medium	0.167	0.160	0.159	0.155	**0.150**
V2_03_difficult	0.187	0.155	**0.153**	0.208	0.192

**Table 2:** RMSE trajectory error on VIODE under dynamic interference. We use “*” to denote tracking failure (no valid trajectory output) or catastrophic divergence. All reported numbers are RMSE ATE (m) after alignment to ground truth using the same protocol. Bold values indicate the best result(s) in each row.

Scenario	Level	ORB-SLAM3	VINS-Fusion	RISE-VIO
City day	0_none	1.940	0.210	**0.129**
	1_low	0.857	0.182	**0.170**
	2_mid	4.486	0.560	**0.216**
	3_high	*	0.510	**0.292**
City night	0_none	*	0.328	**0.262**
	1_low	*	0.371	**0.298**
	2_mid	*	0.457	**0.262**
	3_high	*	0.464	**0.385**
Parking lot	0_none	0.415	**0.102**	0.116
	1_low	0.245	0.138	**0.126**
	2_mid	3.807	0.707	**0.227**
	3_high	4.687	1.135	**0.310**

**Table 3:** EuRoC MAV RMSE comparison of VINS-Fusion, OpenVINS, and RISE-VIO variants. RISE-VIO (a) enables only GNC-DRT-INIT (robust initialization); RISE-VIO (b) enables only GNC-EPnP (robust per-frame outlier rejection); RISE-VIO (full) enables both modules. Best per row in **bold**.

Seq.	VINS- Fusion	Open- VINS	RISE-VIO (a)	RISE-VIO (b)	RISE-VIO (Full)
MH_01_easy	0.180	0.157	0.130	0.121	**0.114**
MH_02_easy	0.105	0.104	0.098	0.079	**0.070**
MH_03_medium	0.143	0.280	0.144	0.163	**0.119**
MH_04_difficult	0.216	0.174	0.163	0.160	**0.148**
MH_05_difficult	0.290	0.262	0.131	0.187	**0.131**
V1_01_easy	0.071	**0.070**	0.075	0.094	0.073
V1_02_medium	0.128	0.261	0.116	0.113	**0.099**
V1_03_difficult	0.170	**0.080**	0.102	0.113	0.098
V2_01_easy	0.142	0.110	0.075	0.063	**0.061**
V2_02_medium	0.167	**0.097**	0.137	0.104	0.102
V2_03_difficult	0.187	0.146	0.166	0.154	**0.132**

**Table 4:** Run-to-run variability of RISE-VIO (full) on EuRoC MAV. RMSE ATE (m) over five replays per sequence; we report mean and sample standard deviation.

Seq.	Mean (m)	Std (m)
MH_01_easy	0.114	0.010
MH_02_easy	0.070	0.012
MH_03_medium	0.119	0.008
MH_04_difficult	0.148	0.021
MH_05_difficult	0.131	0.014
V1_01_easy	0.073	0.006
V1_02_medium	0.099	0.006
V1_03_difficult	0.098	0.002
V2_01_easy	0.061	0.003
V2_02_medium	0.102	0.006
V2_03_difficult	0.132	0.006

**Table 5:** EuRoC MAV relative trajectory error (RTE) over different segment lengths. RTE is evaluated over fixed segment lengths of 3 m, 5 m, and 10 m. Lower is better. The best result in each row and segment length is shown in **bold**.

Seq.	RTE @ 3 m		RTE @ 5 m		RTE @ 10 m	
	**VINS-** **Fusion**	**RISE-VIO** **(Full)**	**VINS-** **Fusion**	**RISE-VIO** **(Full)**	**VINS-** **Fusion**	**RISE-VIO** **(Full)**
MH_01_easy	0.076	**0.071**	0.111	**0.102**	0.180	**0.168**
MH_02_easy	0.071	**0.063**	**0.104**	0.110	**0.186**	0.262
MH_03_medium	0.101	**0.087**	0.146	**0.145**	0.225	**0.224**
MH_04_difficult	0.112	**0.093**	0.166	**0.143**	0.282	**0.254**
MH_05_difficult	0.106	**0.097**	0.179	**0.158**	**0.248**	0.326
V1_01_easy	0.228	**0.221**	0.317	**0.300**	0.303	**0.302**
V1_02_medium	0.135	**0.114**	0.200	**0.135**	0.217	**0.124**
V1_03_difficult	0.101	**0.096**	**0.122**	0.125	0.171	**0.151**
V2_01_easy	0.090	**0.073**	**0.086**	0.099	**0.109**	0.125
V2_02_medium	0.091	**0.068**	0.139	**0.096**	**0.132**	0.153
V2_03_difficult	**0.097**	0.108	**0.121**	0.139	**0.132**	0.167

**Table 6:** Real-time replay performance on EuRoC (MH_05_difficult). System-level cost is measured by wall-clock time, CPU utilization, and peak resident memory. Online latency is reported as the delay between the odometry timestamp and the current ROS time.

Config	Wall time (s)	CPU (%)	Max RSS (MB)	Delay (s) Mean/Median/p95
Benchmark	132.47	112	190.4	0.089/0.080/0.137
Demo	142.63	252	798.4	0.096/0.088/0.142

## Data Availability

The source code for the proposed method is publicly available at: https://github.com/shoulg/RISE-VIO (accessed on 1 April 2026). The EuRoC MAV dataset was obtained from the publicly available benchmark at https://projects.asl.ethz.ch/datasets/euroc-mav/ (DOI: 10.1177/0278364915620033; accessed on 1 April 2026). The VIODE dataset used for robustness evaluation is publicly available at https://github.com/kminoda/VIODE and archived with DOI: 10.5281/zenodo.4493401 (accessed on 1 April 2026). Monte Carlo simulation data generated in this study are available from the corresponding author upon reasonable request.
